# FMDV replicons encoding green fluorescent protein are replication competent

**DOI:** 10.1016/j.jviromet.2014.08.020

**Published:** 2014-12-01

**Authors:** Fiona Tulloch, Uday Pathania, Garry A. Luke, John Nicholson, Nicola J. Stonehouse, David J. Rowlands, Terry Jackson, Toby Tuthill, Juergen Haas, Angus I. Lamond, Martin D. Ryan

**Affiliations:** a Centre for Biomolecular Sciences, School of Biology, University of St Andrews, North Haugh, St Andrews KY16 9ST, UK; b School of Molecular and Cellular Biology, Faculty of Biological Sciences, University of Leeds, Leeds LS2 9JT, UK; c The Pirbright Institute, Ash Road, Pirbright, Surrey GU24 ONF, UK; d Division of Pathway Medicine, University of Edinburgh, 49 Little France Crescent, Edinburgh EH16 4SB, UK; e Centre for Gene Regulation and Expression, College of Life Sciences, University of Dundee, DD1 5EH, UK

**Keywords:** FMDV, Replicon, Fluorescence, Replication

## Abstract

The study of replication of viruses that require high bio-secure facilities can be accomplished with less stringent containment using non-infectious ‘replicon’ systems. The FMDV replicon system (pT7rep) reported by Mclnerney et al. (2000) was modified by the replacement of sequences encoding chloramphenicol acetyl-transferase (CAT) with those encoding a functional L proteinase (L^pro^) linked to a bi-functional fluorescent/antibiotic resistance fusion protein (green fluorescent protein/puromycin resistance, [GFP-PAC]). Cells were transfected with replicon-derived transcript RNA and GFP fluorescence quantified. Replication of transcript RNAs was readily detected by fluorescence, whilst the signal from replication-incompetent forms of the genome was >2-fold lower. Surprisingly, a form of the replicon lacking the L^pro^ showed a significantly stronger fluorescence signal, but appeared with slightly delayed kinetics. Replication can, therefore, be quantified simply by live-cell imaging and image analyses, providing a rapid and facile alternative to RT-qPCR or CAT assays.

## Highlights

• FMDV replication can be studied outwith high disease secure facilities.
• FMDV replicon genomes encoding GFP are replication competent.
• These FMDV replicon systems can be used to study replication by live-cell imaging/image analyses.

## 1 Introduction

**Fig. 1:**
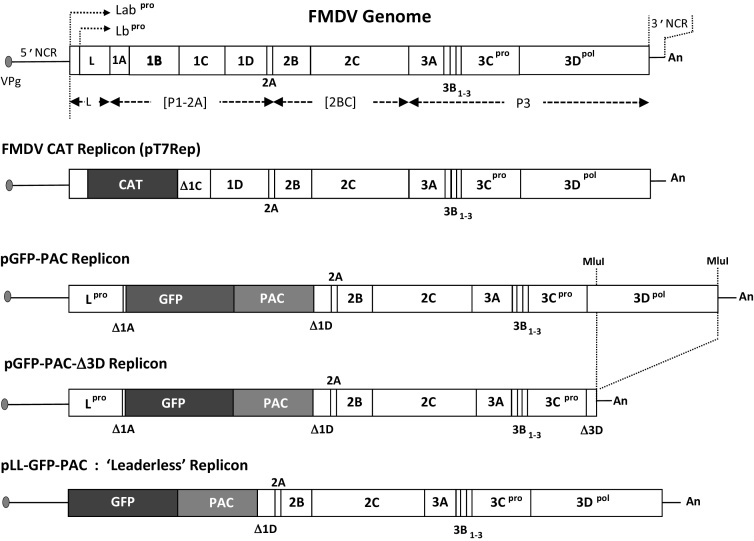
Replicon constructs. The structure of the FMDV genome is shown together with replicon plasmid constructs. Polyprotein domains are shown as boxed areas, together with the ‘primary’ processing products L^pro^ (Lab^pro^ and Lb^pro^ forms), [P1-2A], [2BC] and P3 ([3AB_1–3_CD]). The original CAT replicon (pT7Rep; Ellard et al., 1999, Mclnerney et al., 2000) was modified to re-insert the L proteinase sequences and the CAT reporter replaced with a GFP-PAC fusion protein (pGFP-PAC). This plasmid was modified to create a replication incompetent form by deletion of the 3D polymerase (pGFP-PAC-Δ3D). A replication attenuated form was created by deletion of the L proteinase (pLL-GFP-PAC; similar to that described by Piccone et al., 1995).

Foot-and-mouth disease virus (FMDV: family *Picornaviridae*, genus *Aphthovirus*) causes a highly contagious and economically devastating disease of cattle and other cloven-hoofed animals. The genome consists of single-stranded, positive-sense, RNA of ∼8.5 kb. The genome is covalently linked to the virus-encoded peptide VPg (3B) at its 5′ terminus and is polyadenylated at its 3′ terminus. Naked viral RNA is sufficient to initiate replication when introduced into the cytoplasm since it can act directly as an mRNA. The genome encodes a single open reading frame (ORF) of ∼2300aa. FMDV is unusual amongst picornaviruses in that initiation of translation occurs at two sites giving rise to two different forms of the N-terminal L proteinase (Lab^pro^ and Lb^pro^). The full-length translation product (‘polyprotein’) is not observed, however, due to ‘processing’: a combination of extremely rapid co-translational (‘primary’) proteolytic cleavages by virus-encoded proteinases (reviewed by Ryan and Flint, 1997), and, by a translational recoding mechanism at the 2A/2B site: ‘ribosome skipping’ (Ryan et al., 1999, Donnelly et al., 2001). In FMDV the primary processing products are L^pro^ (cleaves at its own C-terminus), [P1-2A], [2BC] and P3 (Fig. 1). The [P1-2A] product is the precursor for post-translational ‘secondary’ processing by the 3C/3CD proteinases (3C^pro^/3CD^pro^) generating the structural proteins 1A-D, whereas 2BC and P3 are precursors for proteins required for the replication of the viral genome. Host-cell capped mRNA translation is shut-off since L^pro^ cleaves the eIF4G component of the eIF4F cap-binding protein complex (Devaney et al., 1988). FMDV translation initiates, however, using an internal ribosome entry site (IRES) in the 5′ non-coding region (NCR) in a cap-independent manner (Belsham and Brangwyn, 1990). Interestingly, virus could not be rescued from BHK-21 cells transfected with FMDV transcript RNA lacking L^pro^, however viruses containing the “spacer” region between the two L^pro^ initiation codons, produced slightly smaller plaques and grew to slightly lower titres than WT virus. When tested in mice this virus was only slightly attenuated, but more highly attenuated in swine (Piccone et al., 1995, Chinsangaram et al., 1998).

Picornavirus ‘replicon’ genomes can be created by in-frame deletions within the region encoding the capsid proteins. Such genomes are replication competent but cannot encapsidate themselves. They can, however, be encapsidated by ‘helper’ viruses providing capsid proteins *in trans*. In some picornaviruses, however, a *cis*-acting replication RNA structural element (*cre*) is present in the region encoding capsid proteins and cannot be deleted (McKnight and Lemon, 1996): in the case of FMDV, the cre has been mapped to a region within the 5′ non-coding region immediately 5′ of the IRES of the genome (Mason et al., 2002). FMDV replicon systems provide a unique opportunity of studying FMDV replication outwith high-security disease containment facilities, since (a) they cannot form infectious viruses by themselves, and, (b) recombination with circulating FMDV can absolutely be discounted if the laboratory is situated within a ‘disease (virus) free’ region.

Previously, an FMDV replicon based on the genome of FMDV type-O O1/Kaufbeuren/FRG/66 (Forss et al., 1984) was created encoding the chloramphenicol acetyl-transferase (CAT) reporter gene. Most of the coding sequence for L^pro^ was deleted, along with capsid proteins 1A and 1B, and just over half of capsid protein 1C (pT7rep; Mclnerney et al., 2000; Fig. 1). These deletions did not abrogate replicon-derived RNA replication (Mclnerney et al., 2000), an observation consistent with the FMDV *cre* being located within the 5′NCR and L^pro^ not being essential for FMDV replication. This paper describes the construction of a new FMDV replicon system encoding the full repertoire of non-structural proteins which provides a rapid, facile, method of studying replication, based upon live-cell imaging together with quantitation of fluorescence.

## 2 Materials and methods

All plasmids in this report were constructed using standard methods and confirmed by nucleotide sequencing. Restriction enzymes were purchased from Promega (Southampton, UK) and New England Biolabs (Hitchin, UK), whilst oligonucleotides were obtained from IDT Technologies (Leuven, Belgium).

### 2.1 Construction of GFP-PAC replicon (pGFP-PAC)

For simplicity in the construction, unique restriction sites within the 5′ non-coding region (NCR) just upstream of the Lab initiation codon (*Psi*I) and within capsid protein 1D (*Sma*I/*Xma*I) were used. Sequences encoding L^pro^ and the first 18aa of capsid protein 1A plus sequences encoding green fluorescent protein/puromycin resistance fusion protein (GFP-PAC) were initially assembled as a single ORF in construct pJC3mod (unpublished). The L^pro^/GFP-PAC sequences were amplified from this construct using primers JN6 (5′-TTATAACCACTGAACAC**ATG**AATACAA CTGACTGTTTT-3′: *Psi*I site underlined, L^pro^ initiation codon in bold) and JN7 (5′-CCCGGGTGGC ACCGGGCTTGCGGGTCATGCA-3′: *Sma*I/*Xma*I site underlined) and the product ligated into pGEM-T Easy (Promega) following the manufacturer's instructions. The insert was purified following restriction with *Psi*I/*Xma*I and ligated into pT7Rep, similarly restricted, to create pGFP-PAC (Fig. 1).

### 2.2 Construction of a ‘leaderless’ GFP-PAC replicon form (pLL-GFP-PAC)

Sequences encoding green fluorescent protein/puromycin resistance fusion protein (GFP-PAC) were amplified as described above. Primers JN8 (5′-TTATAACCACTGAACAC**ATG** GATATCGTGTCCAAAGGGGAAG-3′: *Psi*I site underlined, GFP initiation codon in bold) and JN7 (5′-CCCGGGTGGCACCGGGCTTGCGGGTCATGCA-3′: *Sma*I/*Xma*I site underlined) and the product ligated into pGEM-T Easy (Promega) following the manufacturer's instructions. The insert was purified following restriction with *Psi*I/*Xma*I and ligated into pT7Rep, similarly restricted, to create pLL-GFP-PAC (Fig. 1).

### 2.3 Replication incompetent (control) replicon form

A replication incompetent (control) form of pGFP-PAC replicon was created by a large, in-frame, deletion within the region encoding the 3D RNA-dependent RNA polymerase (3D^pol^) by restriction of the replicon with *Mlu*I and re-ligation to create pGFP-PAC-Δ3D (Fig. 1).

### 2.4 RNA transcription and cell transfection

Replicon constructs were transcribed *in vitro* using T7 RNA polymerase and transcript RNA used to transfect cells. Baby hamster kidney (BHK-21) cells were obtained from the American Type Culture Collection (ATCC, Teddington, UK) and propagated in Dulbecco's modified eagle medium (DMEM) containing 10% foetal calf serum at 37 °C/5% CO_2_. Plasmid constructs were linearised with *Hpa*I, RNA transcripts prepared using T7 RNA Polymerase (Promega) as per the manufacturer's instructions. Transcript RNA was analysed by 0.8% agarose gel electrophoresis and spectrophotometery (NanoDrop 1000 ThermoFisher, Wilmington, USA). RNA (1 μg) was transfected into cell monolayers (1 × 10^5^ cells/well: 80–90% confluent) using Lipofectamine-2000 (Life Technologies, Paisley, UK), as per the manufacturer's instructions.

### 2.5 Quantitation of GFP fluorescence

Images of transfected cells were captured at intervals between 0 and 24 h post-transfection using an IncuCyte ZOOM kinetic imaging system (Essen BioScience, Welwyn Garden City, UK) housed within an incubator maintained at 37 °C/5% CO_2_. Images were captured from nine regions/well using the 10× objective (data analyses) or 4× objective (animation provided in Supplementary data). GFP positive cell counts and GFP intensities were measured using the IncuCyte image processing software. Values from all nine regions of each well were pooled and averaged across four replicates (Fig. 3, Fig. 4).

### 2.6 Western blotting

BHK-21 cells were transfected with RNA transcripts from pGFP-PAC or pLL-GFP-PAC, cell extracts prepared at 1, 2, 4, 6, 8, 10 and 24 h post-transfection using RIPA lysis buffer (150 mM NaCl, 50 mM Tris–HCl [pH 7.4], 1% NP-40, 0.5% deoxycholic acid, 0.1% SDS). Samples were resolved by 10% SDS-PAGE, transferred to a nitrocellulose membrane (Life Technologies) and blocked for 1 h in 5% PBS-T (PBS, 0.1% Tween 20, supplemented with 5% non-fat milk). The membrane was probed with rabbit anti-eIF4G (1:1000: kind gift of Lisa Roberts) or mouse anti-tubulin (Invitrogen, 1:2000) overnight at 4 °C. The membrane was then washed 3× in PBS-T, then probed with peroxidase conjugated anti-rabbit (Invitrogen, 1:2000) or anti-mouse (Invitrogen, 1:2000) antibodies in 5% PBS-T for 1 h at room temperature. Membranes were washed 3× in PBS-T and antibody binding detected using an EZ-ECL HRP chemiluminescence detection kit Biological Industries, Kibbutz Beit-Haemek, Israel).

## 3 Results

### 3.1 Construct design

L^pro^ cleaves at its own C-terminus: to ensure correct proteolytic processing at this site, the N-terminal 18aa of capsid protein 1A was included between L^pro^ and GFP-PAC, with an additional 5aa from ‘linker’ RE sites created between the 1A sequences and the GFP-PAC fusion protein. To ensure highly efficient processing (‘ribosome skipping’) by 2A at the 2A/2B site, the C-terminal 40aa of capsid protein 1D were included upstream of 2A (Fig. 1; Donnelly et al., 2001). Processing by L^pro^ and 2A produces therefore a [Δ1A-GFP-PAC-Δ1D-2A] product. It had previously been determined that the GFP-PAC fusion protein retained fluorescence and conferred puromycin resistance (the PAC sequences were included for later studies on the molecular mechanisms of virus persistence). This reporter protein allowed live-cell, real-time, monitoring of replication using cell-imaging and quantitation of fluorescence.

### 3.2 L proteinase activity

**Fig. 2:**
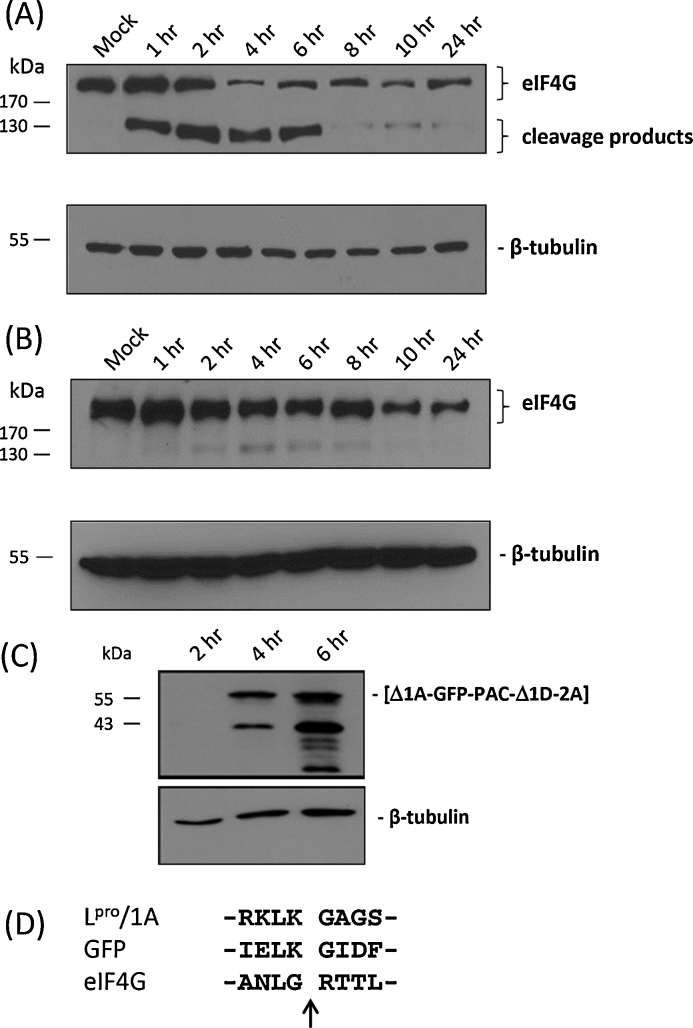
Cleavage of eIF4G and GFP in cells transfected with replicon RNA. Extracts were prepared from pGFP-PAC (panels A and C) and pLL-GFP-PAC (panel B) replicon-transfected BHK-21 cells at the time points indicated. Extracts were separated by 10% SDS-PAGE, transferred to nitrocellulose membranes, and analysed by western blotting with anti-eIF4G (panels A and B), anti-GFP (panel C) and anti-β-tubulin antibodies (panels A–C). The sequence flanking the L^pro^/1A, eIF4G and predicted GFP L^pro^ cleavage sites are shown (Panel D).

The expectation was that a replicon genome could be constructed that would generate a self-replicating mRNA and, since L^pro^ had been restored, eIF4G would be cleaved and the translation of host-cell mRNA shut-off. To confirm the L^pro^ that had been re-instated was active, western blots were performed to determine if the major host-cell protein target of L^pro^, eIF4G, was cleaved. The data showed that L^pro^ was active in the pGFP-PAC construct and had degraded eIF4G (Fig. 2, panel A). Transfection of cells with the leaderless form of the replicon (pLL-GFP-PAC) showed some degradation of eIF4G (Fig. 2, panel B), but with slower kinetics than observed for pGFP-PAC – consistent with the observation that the FMDV 3C proteinase also can degrade this initiation factor (Belsham et al., 2000).

### 3.3 Quantitation of FMDV replicon-derived GFP fluorescence

RNA replication was quantified by the indirect measure of GFP fluorescence. Since transcript RNAs may act as an mRNA, it was critical to determine the intensity of the ‘signal’ fluorescence (increased translation arising from replication of the input RNA) from that of ‘background’ (translation from the input RNA alone in the absence of replication). An additional factor here is that even in the case of replication incompetent forms, translation from the IRES will be relatively enhanced by the presence of an active L^pro^, since translation of host-cell capped mRNA would be shut-off, conferring an advantage for IRES-driven translation.

**Fig. 3:**
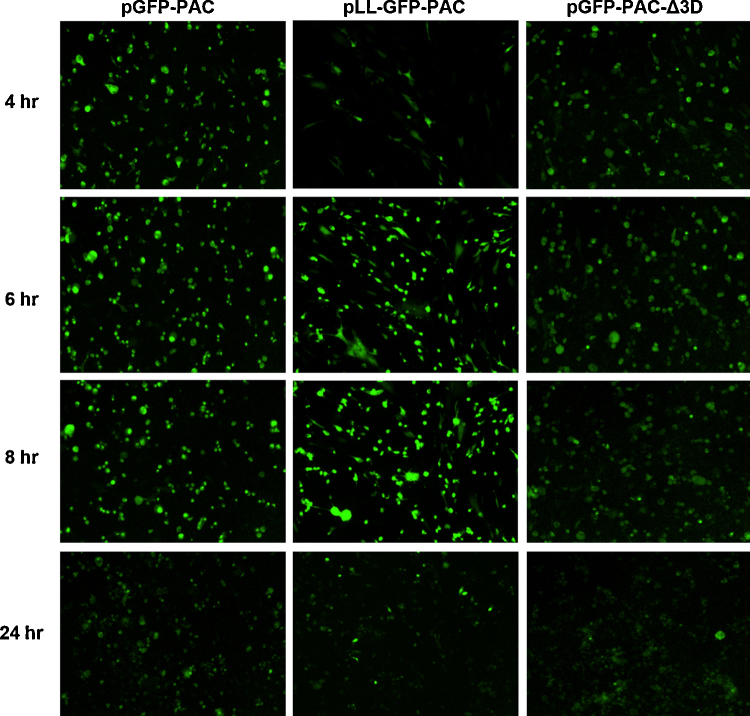
GFP expression in FMDV replicon-transfected BHK-21 cells. Transcript RNAs from the pGFP-PAC, pLL-GFP-PAC and pGFP-PAC-Δ3D replicons were transfected into cell monolayers, and fluorescent images captured at 2 h intervals over a 24 h period using the IncuCyte ZOOM imaging system. A representative of the nine images (captured for each well at each time point) is shown.

**Fig. 4:**
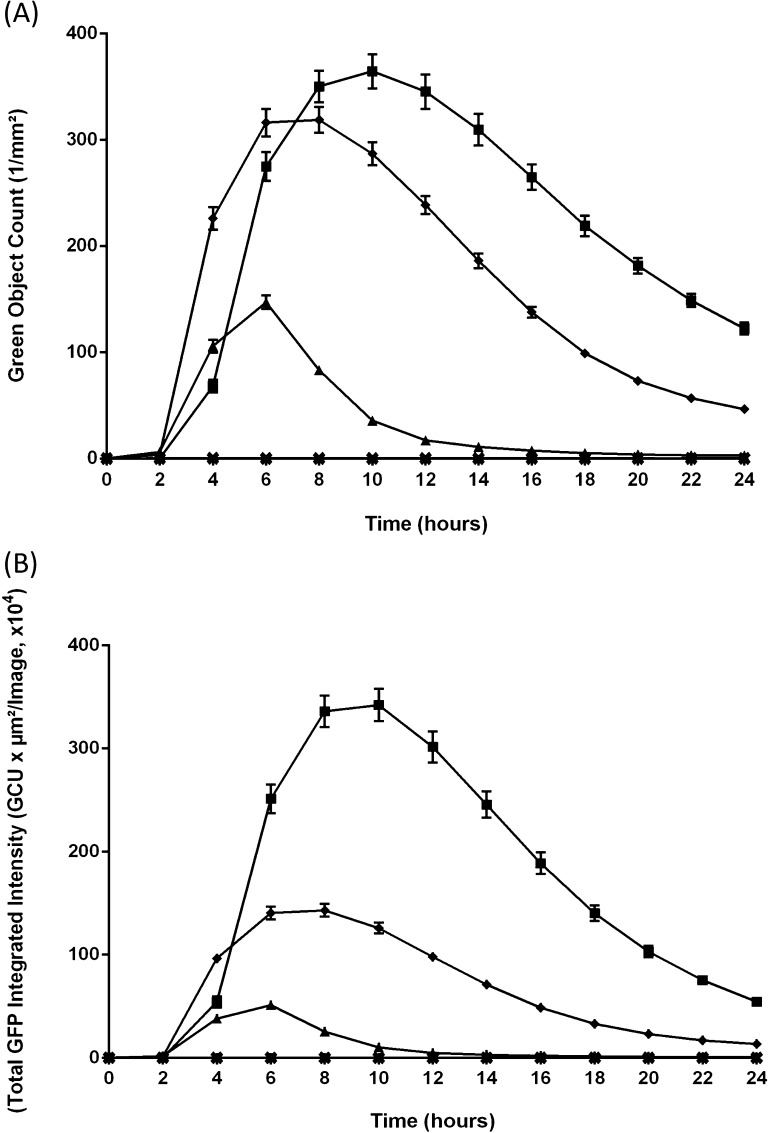
Time course of FMDV replicon-derived GFP fluorescence. Data from mock-transfected BHK-21 cells are shown (×), together with cells transfected with transcript RNA derived from the pGFP-PAC replicon (◆), ‘leaderless’ replicon pLL-GFP-PAC (■) and polymerase deletion pGFP-PAC-Δ3D (▴) constructs. At the time points indicated images were captured and the GFP fluorescence quantified for each replicon construct: data shown as the green object count/mm^2^ (Panel A) or the integrated GFP fluorescence intensity, ×10^4^ (Panel B). Data points/error bars shown are derived from three independent transfections, with four replicates for each transfection.

Although it might be anticipated that a ‘lag’ in the detection of replicon RNA replication *via* a GFP signal may occur in comparison to nucleic acid-based detection systems, the detection of RNA by RT-qPCR, or, in this case indirectly by GFP fluorescence, both require the synthesis of daughter +ve strands from the −ve strand template, plus translation. Detection *via* fluorescence additionally requires the time taken for the post-translational modifications required to generate the GFP fluorophore, although these are relatively rapid. The appearance of the GFP fluorescence signal (∼2 h post-transfection; Fig. 3, Fig. 4) is directly comparable to detection of vRNA replication within FMDV-infected cells by strand-specific RT-qPCR (Gu et al., 2007, Chang et al., 2009). The fluorescence data is presented as the green object (cell) count/mm^2^ (Fig. 4, panel A) and the integrated (total) intensity of the fluorescence signal (Fig. 4, panel B).

Although RNA derived from constructs encoding L^pro^ and bearing deletions within 3D^pol^ (pGFP-PAC-Δ3D) are unable to replicate, L^pro^ is produced as a result of translation of the input RNA. Thus upon transfection, it was predicted that host-cell capped mRNA translation would be reduced and the translation of the replicon RNA enhanced, since in this case translation is IRES-driven and not dependent on the presence of intact eIF4G. Consistent with this prediction, GFP fluorescence was detected on transfection of RNA derived from pGFP-PAC-Δ3D albeit at a reduced level (∼2-fold lower at the signal peak) when compared to the replication competent form (pGFP-PAC; Fig. 4, panels A and B).

Although the number of green cells is similar for the wild-type and leaderless replicon constructs, the intensity of the fluorescence signal is notably higher for the leaderless construct, albeit with slightly delayed kinetics in both cases (Fig. 3, Fig. 4). This was surprising since L^pro^ shuts-off host-cell cap-dependent mRNA translation and it was expected that deletion of L^pro^ would reduce translation of virus proteins since the replicon RNA would now compete with cellular mRNAs for the translational resources of the cell. Furthermore, western blotting of replicon transfected cell extracts with anti-GFP antibodies showed that the [Δ1A-GFP-PAC-Δ1D-2A] processing product was somewhat degraded in cells transfected with the pGFP-PAC replicon encoding a functional L^pro^ (Fig. 2, panel C), but is not degraded in the case of leaderless form (data not shown). The estimated molecular mass of the major degradation product (∼42 kDa; Fig. 2, panel C) suggests a proteinase cleavage site within [Δ1A-GFP-PAC-Δ1D-2A]: examination of the GFP/PAC sequences suggested a potential L^pro^ cleavage site at a lysine^127^–glycine^128^ pair within GFP, which would produce a cleavage product of 41.6 kDa (corresponding well with the molecular mass of the observed cleavage product), the site mapping to the C-terminus of β-sheet 6 – a surface feature (Ormö et al., 1996). No potential 3C^pro^ cleavage sites were detected within GFP, and no potential L^pro^/3C^pro^ cleavage sites could be detected within PAC. An alignment of sequences flanking the L^pro^/1A, eIF4G cleavage sites (Strebel and Beck, 1986, Kirchweger et al., 1994) and the predicted GFP site are shown in Fig. 2, panel D. The cleavage of [Δ1A-GFP-PAC-Δ1D-2A] by L^pro^ is much slower than eIF4G, the cleavage product only appearing at ∼4 h post-transfection, by which time eIF4G is largely degraded.

These data showed that (i) fluorescence started to decline ∼8 h after the appearance of the signal (although the half-life of GFP is ∼24 h), (ii) the analyses of eIF4G degradation showed this protein began to re-accumulate ∼6 h post-transfection, the cleavage product disappearing ∼8 h post-transfection and (iii) microscopy analyses showed a progressive change in the morphology of transfected cells from the typical BHK-21 elongated fibroblastic to a rounded-off form (Fig. 3) – typical of the cytopathic effects observed during virus infection: changes which occurred prior to many cells detaching from the plastic and undergoing necrosis. Taken together, these data show that the analyses ∼8–10 h post-transfection onwards are increasingly those of adherent, non-transfected, cells.

## 4 Discussion

The objective of this work was to generate an FMDV replicon system that could be used outwith high disease secure facilities, but also to use a replicon genome form that could be readily converted into an infectious copy (deletion of sequences encoding GFP-PAC and insertion of those encoding capsid proteins) within an appropriate containment facility. The (indirect) measure of replication *via* GFP fluorescence would facilitate screening studies (mutations/insertions/deletions, virus/host-cell interactions, *etc.*), observations which then could then be verified using virus rescued from the corresponding infectious copy. The *Aquorea* GFP reporter protein used in this study showed degradation by FMDV L^pro^. The potential for the use of such a fluorescent reporter protein was, however, demonstrated by the much stronger signal obtained from the replicon form lacking L^pro^ and experiments to either mutate the putative L^pro^ cleavage site within *Aquorea* GFP to destroy this site, or, the use of an alternative fluorescent protein that is not degraded by FMDV L^pro^.

In summary, these data showed (i) the fluorescence signal intensities could very clearly distinguish between the replicating/non-replicating replicon genomic forms (even with some degradation of GFP by L^pro^), (ii) such a system could be further developed to measure changes in replicative fitness between wild-type and attenuated forms, and (iii) live-cell fluorescence imaging provides a facile method of screening/quantifying FMDV replication – directly comparable to strand-specific RT-qPCR.

## References

[bib0005] Belsham G.J., Brangwyn J.K. (1990). A region of the 5′ noncoding region of foot-and-mouth disease virus RNA directs efficient internal initiation of protein synthesis within cells: involvement with the role of L protease in translational control. J. Virol..

[bib0010] Belsham G.J., McInerney G.M., Ross-Smith N. (2000). Foot-and-mouth disease virus 3C protease induces cleavage of translation initiation factors eIF4A and eIF4G within infected cells. J. Virol..

[bib0015] Chinsangaram J., Mason P.W., Grubman M.J. (1998). Protection of swine by live and inactivated vaccines prepared from a leader proteinase-deficient serotype A12 foot-and-mouth disease virus. Vaccine.

[bib0020] Chang Y., Zheng H., Shang Y., Jin Y., Wang G., Shen X., Liu X. (2009). Recovery of infectious foot-and-mouth disease virus from full-length genomic cDNA clones using an RNA polymerase I system. Acta Biochim. Biophys. Sin. (Shanghai).

[bib0025] Devaney M.A., Vakharia V.N., Lloyd R.E., Ehrenfeld E., Grubman M.J. (1988). Leader protein of foot-and-mouth disease virus is required for cleavage of the p220 component of the cap-binding protein complex. J. Virol..

[bib0030] Donnelly M.L.L., Luke G., Mehrotra A., Li X., Hughes L.E., Gani D., Ryan M.D. (2001). Analysis of the aphthovirus 2A/2B polyprotein ‘cleavage’ mechanism indicates not a proteolytic reaction, but a novel translational effect: a putative ribosomal ‘skip’. J. Gen. Virol..

[bib0035] Ellard F.M., Drew J., Blakemore W.E., Stuart D.I., King A.M. (1999). Evidence for the role of His-142 of protein 1C in the acid-induced disassembly of foot-and-mouth disease virus capsids. J. Gen. Virol..

[bib0040] Forss S., Strebel K., Beck E., Schaller H. (1984). Nucleotide sequence and genome organization of foot-and-mouth disease virus. Nucleic Acids Res..

[bib0045] Gu C., Zheng C., Shi L., Zhang Q., Li Y., Lu B., Xiong Y., Qu S., Shao J., Chang H. (2007). Plus- and minus-stranded foot-and-mouth disease virus RNA quantified simultaneously using a novel real-time RT-PCR. Virus Genes.

[bib0050] Kirchweger R., Ziegler E., Lamphear B.J., Waters D., Liebig H.D., Sommergruber W., Sobrino F., Hohenadl C., Blaas D., Rhoads R.E., Skern T. (1994). Foot-and-mouth disease virus leader proteinase: purification of the Lb form and determination of its cleavage site on eIF-4 gamma. J. Virol..

[bib0055] Mason P.W., Bezborodeva S.V., Henry T.M. (2002). Identification and characterisation of a *cis*-acting replication element (*cre*) adjacent to the internal ribosome entry site of foot-and-mouth disease virus. J. Virol..

[bib0060] McKnight K.L., Lemon S.M. (1996). Capsid coding sequence is required for efficient replication of human rhinovirus 14 RNA. J. Virol..

[bib0065] Mclnerney M.G., Andrew M.Q., Smith N., Belsham G.J. (2000). Replication-competent foot-and-mouth disease virus RNAs lacking capsid coding sequences. J. Gen. Virol..

[bib0070] Ormö M., Cubitt A.B., Kallio K., Gross L.A., Tsien R.Y., Remington S.J. (1996). Crystal structure of the *Aequorea victoria* green fluorescent protein. Science.

[bib0075] Piccone M.E., Rieder E., Mason P.W., Grubman M.J. (1995). The foot-and-mouth disease virus leader proteinase gene is not required for viral replication. J. Virol..

[bib0080] Ryan M.D., Flint M. (1997). Virus-encoded proteinases of the picornavirus super-group. J. Gen. Virol..

[bib0085] Ryan M.D., Donnelly M.L.L., Lewis A., Mehrotra A.P., Wilkie J., Gani D. (1999). A model for non-stoichiometric, co-translational protein scission in eukaryotic ribosomes. Bioorg. Chem..

[bib0090] Strebel K., Beck E. (1986). A second protease of foot-and-mouth-disease virus. J. Virol..

